# Supplementary material for: Development and application of 14 microsatellite markers in the burying beetle Nicrophorus vespilloides reveals population genetic differentiation at local spatial scales
Source: PeerJ. 2017 May 2;5:e3278. doi: 10.7717/peerj.3278 (PMC5417058; doi:10.7717/peerj.3278)
Supplement: Supplemental Information 1 [file peerj-05-3278-s002.pdf]

Supplementary information: *Nicrophorus vespilloides* microsatellite details

1. Microsatellite sequences

Nicrophorus vespilloides sequences containing the developed microsatellite markers. **Red** highlights the microsatellite position in the sequence and shaded in blue are the forward and reverse primers, as identified by msatcommander (Faircloth, 2008). **Raw data:** *N. vespilloides* genome (NCBI Bioproject number PRJNA284849; Cunningham et al., 2015) and transcriptome (NCBI Bioproject PRJNA285436; Parker et al., 2015).

- **gi|941349225|gb|LJCH01000619.1|Genome –** MsatA -138bp

TACATTTCATACGGCCAGAAACCCACCGAGATGATCTTAGACAATAAGCAAGAATATGATCTCAGCATAC
CGTATCAAAGCTACGGCGTGCAGAATTACCCTCCGATGCAACAGCAGCAGCAGCAGCAACAACAACAGC**A**
ACAACAACAACAACAACAACAACAACAGCAGGTTGAAGAACGCAGCGATGTCGACAGCGACGTCGACACC
AGAGAGTTCGACAAGTATCTCAAATTCACCAACCAGGAGTCCAACATCATCGACTCAAATCACAACTATC
ACAGCAGAAACGACGTGATCAATTCGAATATGACATACAACTTCCAAACTCAACATCCTTCCGTTATCCT

- **gi|941348249|gb|LJCH01001595.1| Genome –** MsatD -201bp

GTTGTCATATTTGATCAAATTGAAGCTTATAGTTTTATCTTCAATTTATACATTAGTATAATTAATTAAC
TAATTGAATTTATACTGGAAACTAGCTAAGAATGCAGATGATTGATTTCAATTGCAAATTACGTGCGGTA
ATGAGGCGCGTTTAAAGGAA**AACAACAACAACAACAACAACAACAACAACAAC**AAAAAAGAACGCATTTG
CAAAGGGTGTTTAATTGTGTTCGCGTATTTTATGTGCAGAGCTTAATTTGGTGCGCGCACAAGAGGATGA
AGAGGATAAGTAAATATTATGCATCGAGCGCTAAATAGGGAGCAGGGCGTAAAAGGATGGCAAGACGGCA
AGCTGAGAGATCGCGATAGATAAAGCTTGCAAATCGAAATGGAAAACCGCGTTCGTCCCCCTTTTTAGC

- **gi|941349747|gb|LJCH01000097.1| Genome –** MsatJ -303bp

GAGGTGAGTAAGAGGACAATAAATATATGTTCGTGGATGGGATTGAATTGTAGGTAAGTAATTCGTGGGT
CCACAGAGGATGTGTGTGTGTGTGTAGAGTGGACGGGCACTGGAATGCACAAAAAACAACTTTATATTGT
ATAATATTATCATTTTTATGTCGATGTTTGAGAGCGTTTAACATGGAGTATAAATCGTCTAGCTCGGAGA
GGTGTTGTGTGTTGTACCACGAGCATGTGACAAGCTTCAATAGAGACATTTCCCCCATGTCTCTCTGCCT
GCCTCTCGCACAACTCTC**TTTCTTTCTTTCTTTCTTTCTTTCTTTC**TCTCGTTTTATTAACTCGAAATTT
AAGCGAAAGAACTCGTCTCGTCCTCGTCTTCAACTCGTCCAGGTATGTCACGGGGCAAGGCCAGATAAGA
TATGGACGGTATCGGACGAGTTTATCCGTGTGTATGTGTATGCACATACCCTAATATCGTCGGGCGTAAA

- **gi|941349781|gb|LJCH01000063.1| Genome –** MsatM -373bp

TTTGATGGGAGGATGGGATTTCGGGGGGTTTTTATTTTGGGGTGTAAGGTGTCGAGGTAGTCGCCGTTTT
TCCATTTACCATCGATGGATTGGACGGCGGCCGACCTCGCATCCGCAGCCACAGCACATGCCAGCAACCC
ACAAAGAAGCAACTCAAGAGTATATATAACAAAGCGAGAGCGGGATGAGAGCGAACAAGAATCATATATT
ATATAAAAATAATAATAAATAAAAATAAAAACAGTGAGAGAGAGAGAGATAGAGAGATAGTGAA**AGAGAG**
AGAGAGAGAGAGAGATAATGGTGATCGAGAAGAAGAGATGATAGAGAGTGAGAAGAATTGAAAGATATTG
ATATATAGATTGATTTGTTTGATAGAAAGAGAGAGATAGAGAGTGTGTGTAATTTGTGTGTTAGATCTTT
TGGTGTCGTATTGGGGGTTACCTGGGGATCGGGGAGGCTAAAAGAACTTAATGTCGATGTCTCCAGGTCG
GGACTTGTGGTATGTGTAGATGTGCGTCATGAGCGAGTTGCGAGAGCAGTACACGCGCTCGCAGATGACG
CAACGGTACTCTTCCTGCCGTTCCGCGTGCTTGTCCGCGATGTGTCGCTTTAGGGACGCCTTTGAGCACA

- **gi|941349457|gb|LJCH01000387.1| Genome –** MsatQ -428bp

TGTAGTCTTCTCTCTCAGGTTAGTGCAATAATTATATTGACTAACAGAGAAGGCCCAAAAGTTCACCCAT
CTTCCATTGTCGTATGCGGCTTTGATATCCAGGAATTGTTGGCAGA**TTATTATTATTATTATTATTA**TTC
ACAATTATTGATACTATGCAAAATGCGGTACAATGTGTAAAGTGGGCTGCATAGCGCAATTACAATTTGC
AAAGTATTTTAGGCAGATTGAGTTTACTTTTACTGTCTTTTTGAATAATATTGCTAATAATTATTTCATG
ACTAATTAGTATAATTATTAGAGACAACAAAATGTATTTATTAATTTGAAGATTATTGAGCTAAAAAGCC
ATAGTATTTATTAAAATAATGTTGTTACTACGTGCTTAATGAAATTACATCATGTAAAGATAACATGTTG
CGCGATCCTGCTGGTCGGTCGGATTATGTAACGTCCTGTTCGCACTTGTATGTTTCATATGGAAAGATA
AGGAAGGAGAGCGGAATCTGAATCGATTTGCACCGGGCAACTAATGTCTTGAACGTAGAGGATCCGTGAA
GGAATTCCATATTTAATAGAAATATTTGCATATTTGATGTTCTTACATGCGCTTTAGACCAGTTATTTCT

- **gi|941349837|gb|LJCH01000007.1| Genome** – MsatB -158bp

TCCCTCGCTAATTTTTCATGCTTTCTTCTTTTAGATTTTTTTTTATGATTTCGTTTAATCAGTCGAGGTT
GCTCCGTGGAAGGCCGTTGTTTCCGGTTGTTTGCGAGCGACGGCTTTACCGTTTGTTAGTATGTGTGTGT
GTGCAGCAGAAGCATGTCTCATTACAGA**ACACACACACACACACA**TACACATATACATGGTGAGTGTGTG
TTAGTGTCGTGTTCACGGCCGTTTAACTTCGAAAGGGCCGTTGGAAACGAGAAGAGAAGCCCGGAACGAC
TCCAGGGCCGACCTCGCAGGCAGCAGAGCAACCCTCCTCCGCTCTAATATACAGTCTCAAAGCGACGGGA

- **gi|941348343|gb|LJCH01001501.1| Genome** – MsatF -216bp

ATGACATAAAAATGATTAATAACGTTTACGCGAGGTCCGACACGGCGCTGCACAAATATATCGGAAGGGT
TAAAACCCCCGCACACCGAGGGGCGGCAGCTCGCATTCTTTTTTTTTAAAGGGTTGGGAGGTTGGCAGCA
GTGGTAGATTTAAATGCGTTACGTTGTGCCGTTGTTGTTGATGTAGCAGAACTGCGCTTCAAATAAATTT
GCAAAACACCACAAACAAAAATAAATATAAATATAAAATCGCGTGCATCAAAAC**ACACACACACACACAC**
ACAAATTCACATGTTTTTGCTTTGTGTATTTCGTGCACGTGTATGGATCGTGTTTGTCCTCACGGTCGCA
TCATCCAACCCCCCCACCCCCCTGAGCCCAATTATTTATACAACAATATAATTGATTGGAAAAATAATGG

- **gi|919344888|gb|GDKQ01000971.1|Transcriptome** – MsatI -286bp

GCACGCCTTAACACCGGCATCGGATGAGGCGTAGATCCTGATCACCGGAACCCTCTCGACCACCTCACCC
CTGAACTCGGAATTGAAGACGTCCAAGTCGTGCACGGGTTTGGCGGTGTAAAAGTCCATGCAGTTTATGC
GAAAGCTCGCCA**TCTCTCTCTCTCTCT**NCTTCACCGGTCTCCTTAAAACATGTTGTTCAGAAATTCCTCG
AAGCCGTAAACAAACAACACCGACAACAACAACAACAACGAAAACATTGTCAAAAAATTTGCTTGTTGCG
CACTGGANCCAGTCCTGCCAACCCGGCATAAACCCGGGAATTCGCACAAGCAAAAAAAAAAAGGAAATTC
GTTGGATGAGCGGGGATTTTCTCTTCGTACGATTGGTAGAACCGTTTGTTATTTTTTTCTTGAACGTTTN

- **gi|941349386|gb|LJCH01000458.1| Genome** – MsatP -410bp

ACCATAGAAGTTTTTATGTCATATGTCACGTATAGTGCGGATGCAGCTTAAACCATTAGGAATAATGAAG
AAAAATAAATGCAGATACTATAAAATGTTTGGTGATGCAATTGTGAGGCGATTGCAGCCCTTAGCGTATC
ATTTACGTGGTGGTTCCGCCGCGAAGATCTGAAGTTGCGCAACTCACTTACGGATTAAAGGTGGAAATCG
TGCAGGAATTTATGACCCGACATTAGTCAGTCGCAGTTGCGGCCTGGCGCAGGATTAGGACTTCGAGGAA
GAGAAGAAGCGAGCAGCGAGATTCGAGATCGGGGATGAATGTGGACGATGCAGCGCTGAAAAATTTGCAC
GTTTCGAATCATTTCGTTCTTGTG**ATGATGATGATGATGATGATGATG**GTTGTGATGATCTTGCCGTGCG
TTTCCTTTTGATAAGACCGTCAACAAAAACAACCTTCGGTTGGGATTAAAATAGCGGCCGCGTGATGATG
TTACATCGTCTGCCAACCGGAGAGACTATATGCATGTTTGTGCATGTATATACATATGGATGGATGAATG
TGTACGTGCCGCGTTCGTTGCGGCGTTTATGCTTGTAGCAGAGGCGAGAGCATCCTGGCCTGGCCTAGAA

- **gi|919338980|gb|GDKQ01004844.1|Transcriptome** – MsatE -201bp

NNNNNNNNNNNNNNNNNNNNNNNNNNNNNNNNNNTTGAAATGCGGTGGTCTCGTCGGTCTGGATGGTCGGAT
GGATGGATGGAGAGTGGCCTGCAAAAAAGGGGGAGCCGAAGACGCAGACTCCGCACGCTCTCCGCTACAG
TCCGNC**ACACACACACACACACAC**GCACACGTCTCTGTCGCCGTGTTTGCGCGGGCGGCGCCGAAAGAGATA
CATACAATGATCGGGCGTCGCGACGCCTCGCTCGCTCGCCGCCCTTTCGAAACCATCAACCACCCCGACG
TATCCACACATTTAATTCACTCCTCCGAATGAGGAGGGCGTCAATCGTAAATCTCAATTACGGTAATCAA

- **gi|919330077|gb|GDKQ01010587.1|Transcriptome** – MsatG -224bp

CAGCAGCTTCGCGCCGTTTCGCCCATCCTCGTACCGCTTCGCAGTGCAGTGGGGACGTCTCCGAATCGTT
CGTGTGCGTGTTTCTACCTCAGTTTGATCCGGAACGAGTCGGAGTGTGGTCGTTGCGCTCTATCTCACAC
GTGTGTGTAAATAGTGT**ATATATATATATATAT**NGGAGAGGAAGTTGTGTGTACAGCGCGCGCATAGTGT
TCCATATACGAATCGCACGCTCTATGCTAATGGACGTCTAAATTTTCAAAATGGCTGCCGATTCGGGTAT
GGATACGTGCCCATCACCAGAGATCGCGGACTCCAGGAAGAGGCCGCTCGACGGGGACACCGAGAACGGA
GACACGAAGCGCTCGCACTTCAGCACAGGTGGTGATGGAACATACCACTTCANTCGCACAACTGCAGAAG

- **gi|941349834|gb|LJCH01000010.1| Genome** – MsatH -283bp

CGAGTAATCCTGGATAAAGAAAGAGAAGAAAAAAAAGCAAACCCAATTTACGAACATCTTATTGGGGGAA
ATGTACAAAAGTCTTACGTGCGTGTGCGAGAGAAGCTCCTTCATGATGAAGTTGTCGTAGATGTCTCGTG
CCTGCTTCATCCGTTCGTCCGCTCGTTCCAGTTTCTCGTACTGTTTTATCTGTGAGAAGCAAAAACGGAC
AATGAGAGCGCATCTTGTCGGAGAATCGCTTAAAACCCGGACGCGCACGTACAACATCCGGTGCAAAGAC
AGACAGTAAGTG**AGAGAGAGAGAGAGAGAG**ATACAGAGCGTATTATTACGCTCATCTAGCAAACGTCAGC
GAATCCTGGACAATCATCATCATTATTGTTGTTATGCCAGCCACCACCTTCAAACTGGGTCACACACAAC
AAACACACACAAACATACAATACAACAACCCCATCTCTCTCTCATCTGTCGCGCGCGCGAAATTCAATTT

- **gi|941349831|gb|LJCH01000013.1| Genome –** MsatK -334bp

CCTCTTAAATAGAGATTTAGTTTATGTTTATCCAGGAAGTGGCATTTAGGACCCATAAAATAATTTCTCC
ATGCTCTCATTCTCCCAAACGCAATTACGAAAATAAATAACGGTTTATGCATTAAAATTATATAAATGTC
TCGTAACGCCGCGATTTATTGCGCTCGGTTAGAGACCAAAAAGAAGAGATAGAAAAAAAAACGATCAAGA
GTAAAAGATAAGTTTTGGGCTGTAAGGGCCGTGATATCCATAAAAATGATTCCAAGTGCATTCATATTGT
ATCATGGGTAGGGGTG**AGGAGGAGGAGGAGGAGGAGGAGG**AACAGGAGGCGGGAAGCGATATAATCCTTC
GGCTATTCAAGCCAAGGGCAATAATTCACTTTTATGGACAACTTATGCGCGTCCACGGGAACGGGAGCCA
TCGCTGTGCTACTCATAGGGGAACCCCTGTCCTTTATACACCGGTATGAATTTTAATTCTGGGCCGGGCG

- **gi|941349840|gb|LJCH01000004.1| Genome –** MsatO -395bp

TTAATCGGTTATAGTTGAGTCCACCCAGCTATCTTCGATCAATGAATCAGTTATGCCTTATGCCAATTAA
CGCGTCGAGGATTAATTGATTTTCAGTGTAAGTCTAATTAAATTTTCGCGCACTCCTTC**TTCTTCTTCTT**
CTTCTTCTTCGACGGCGACGAAGGGGAAAAAGTGCATTGCACCATTCAATACCGGGAGGAAACTGTTGTT
TTTTTTGCTTGCTTGTCTGTTTGTTTCAGTTTGCGCTTCTTTCTGTTCTTGTTCATATTGTAAATACGAT
TTCGCGTATAATTAATGGACTTTTAAATGCTCCGGTTGGGAGCGGTCGTAAGCAGAAGGAGGAGGAAGCG
GAATAATGAATACGACGACCTACACAAACTGAAAACCCGTCCTCGTGAGGTAAAGAGTCCAAAAAATGTA
TCATCGGAATCCACCAGTCGCACAGGTAACGATGATGATAATATCGATGCATTCGATACGTTTCCGGATC
GGGTAACGTTGGAAACCGTACCGGATCGAATCCGAAATCGGAAAGGCACGGTTCTTCTTCTGCTGTCAAC

2. BLAST annotation

Table S1. Summary of the BLASTx searches using the sequences containing the microsatellite markers against the NCBI nr database.

	BLASTx	Bit-score	e-value	Query cover	Identity
Nvesp_A	PREDICTED: putative transcription factor SOX-15 [Nicrophorus vespilloides]	170	3e-47	99%	100%
Nvesp_D	No significant similarity found				
Nvesp_J	No significant similarity found				
Nvesp_M	PREDICTED: broad-complex core protein isoforms 1/2/3/4/5-like [Bombus terrestris]	121	5e-32	26%	98%
Nvesp_Q	No significant similarity found				
Nvesp_B	No significant similarity found				
Nvesp_F	No significant similarity found				
Nvesp_I	PREDICTED: DNA polymerase zeta catalytic subunit [Nicrophorus vespilloides]	110	2e-25	36%	100%
Nvesp_P	No significant similarity found				
Nvesp_E	No significant similarity found				
Nvesp_G	PREDICTED: RNA-binding protein Nova-1 isoform X1 [Nicrophorus vespilloides]	100	7e-22	33%	100%
Nvesp_H	PREDICTED: G protein-coupled receptor kinase 1 [Nicrophorus vespilloides]	74.7	1e-12	25%	88%
Nvesp_K	No significant similarity found				
Nvesp_O	No significant similarity found				

Pink: alignment score 80-200; Green: alignment score 50-80

3. Chromatographs

Representation of two random Peak Scanner v.1.0 (Applied Biosystems) chromatographs per genotyped multiplex.

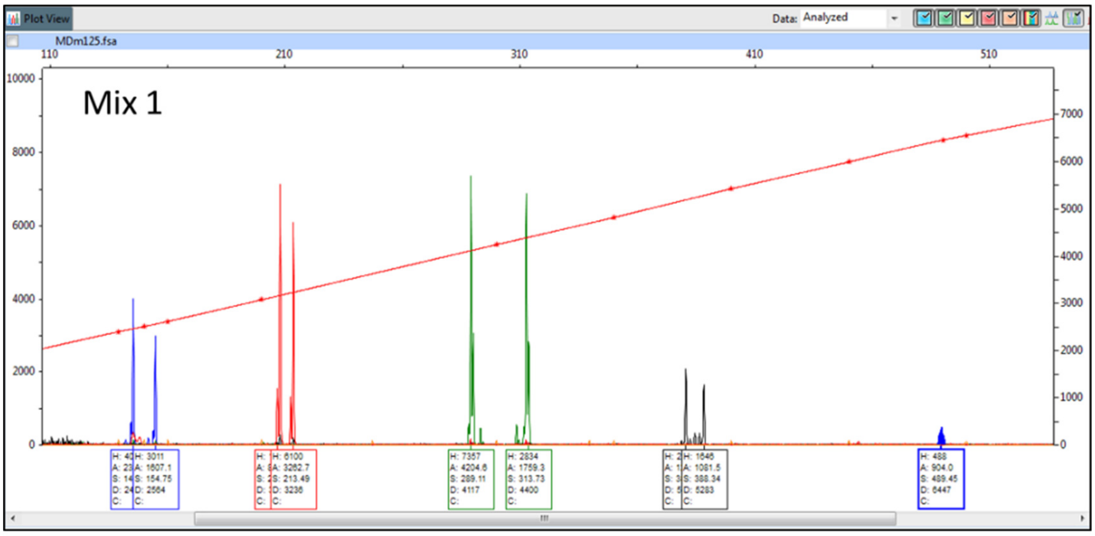

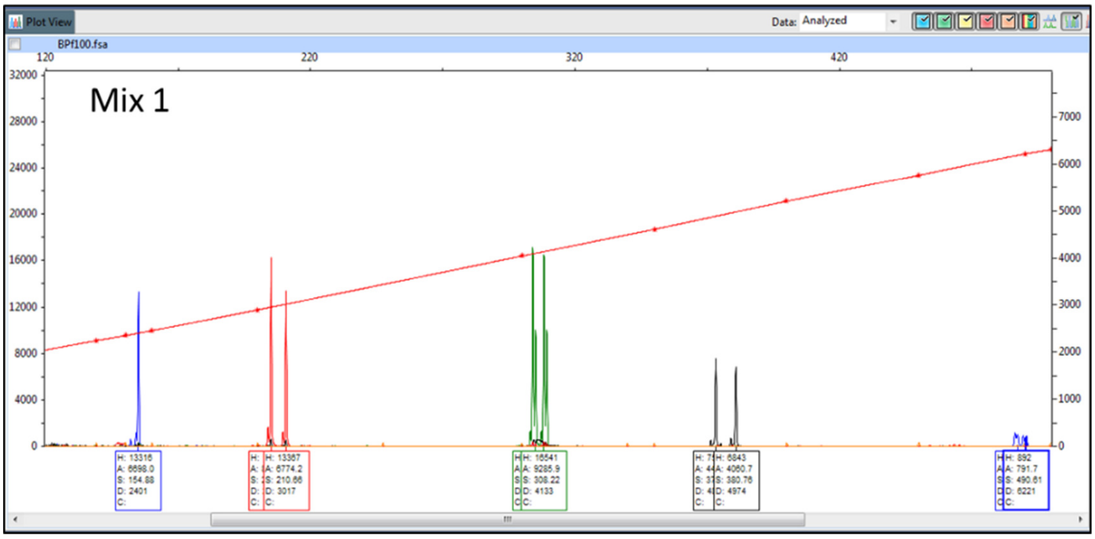

Mix 1: Blue: Nvesp_A; Red: Nvesp_D; Green: Nvesp_J; Black: Nvesp_M; Blue2: Nvesp_Q

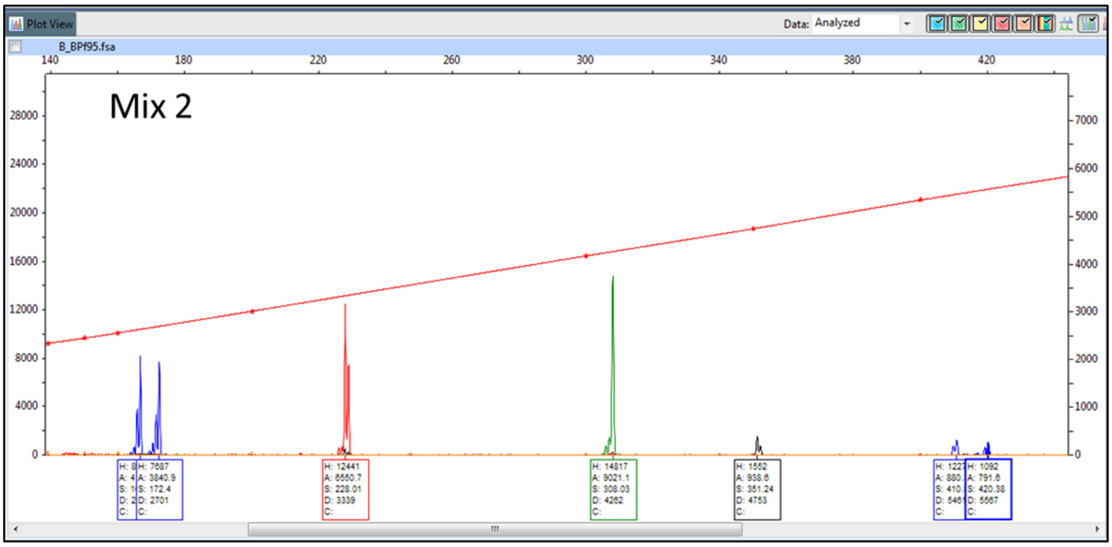

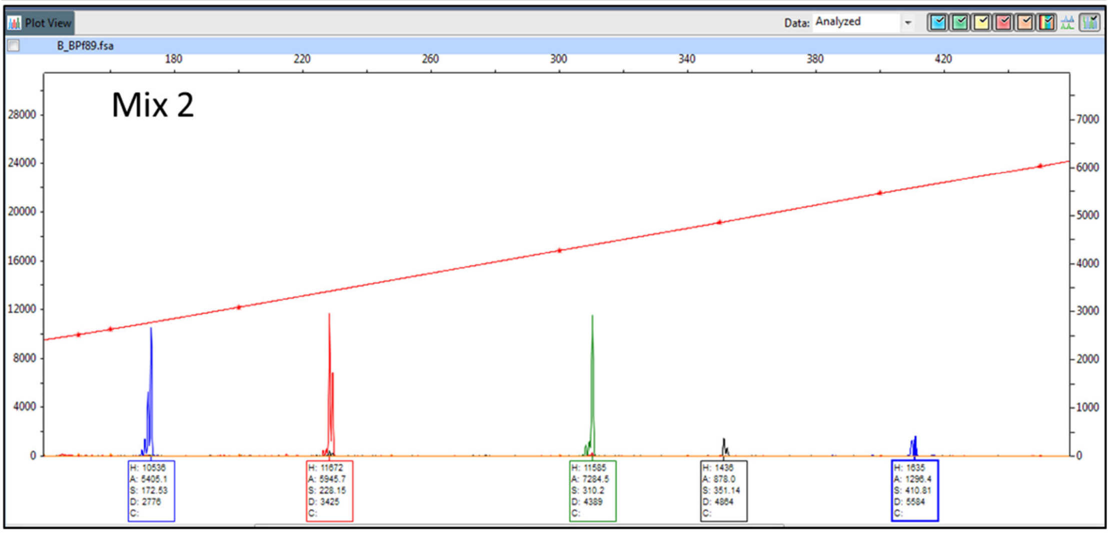

Mix 2: Blue: Nvesp_B; Red: Nvesp_F; Green: Nvesp_I; Black: not used; Blue2: Nvesp_P

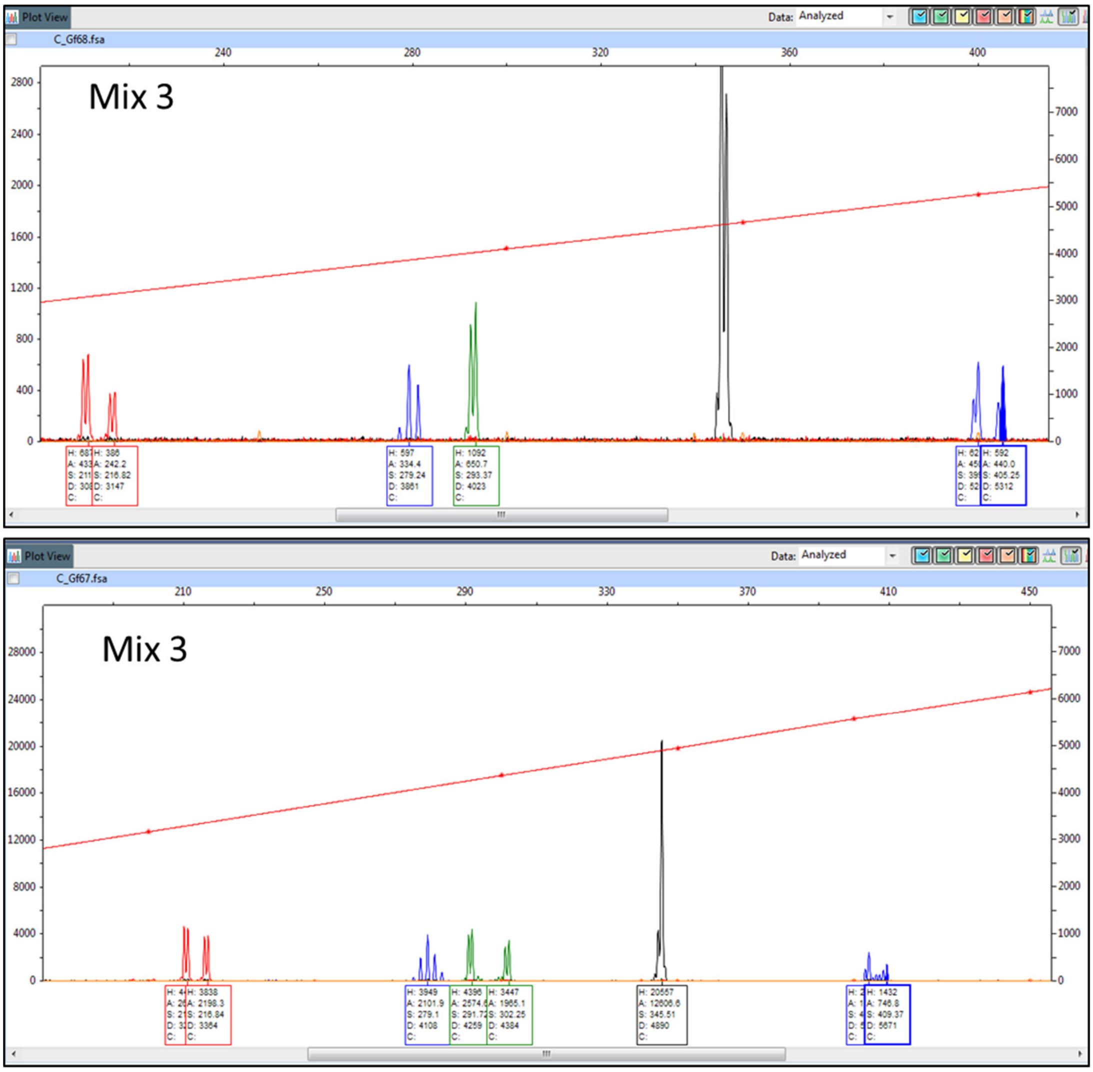

Mix 3: Red: Nvesp_E; Blue: Nvesp_G; Green: Nvesp_H; Black: Nvesp_K; Blue2: Nvesp_O
